# Fixation behavior in macular dystrophy assessed by microperimetry

**DOI:** 10.1007/s00417-018-4006-9

**Published:** 2018-06-09

**Authors:** Wei-Yu Chiang, Jong-Jer Lee, Yi-Hao Chen, Chih-Hsin Chen, Yung-Jen Chen, Pei-Chang Wu, Po-Chiung Fang, Hsi-Kung Kuo

**Affiliations:** Department of Ophthalmology, Kaohsiung Chang Gung Memorial Hospital and Chang Gung University College of Medicine, No.123, Dapi Rd., Niaosong Dist., Kaohsiung City, 83301 Taiwan

**Keywords:** Fixation behavior, Macular dystrophy, Microperimetry, Preferred retinal locus

## Abstract

**Purpose:**

To investigate the fixation behavior in macular dystrophy using microperimetry.

**Methods:**

This retrospective study included patients with macular dystrophy and unilateral macular pucker. Macular dystrophic eyes were compared based on fixation within or outside of the atrophic region. The normal fellow eyes in patients with unilateral macular pucker formed the control group. Clinical and demographic characteristics of age, sex, best-corrected visual acuity, spherical equivalent, and fixation behavior (which included foveal mean sensitivity (MS), fixation MS, MS improvement, stability, centrality, and eccentric distance of fixation) were analyzed. A total of 58 patients were recruited, comprising 29 eyes of 29 patients in the macular dystrophy group and 29 eyes of 29 patients in the control group.

**Results:**

Compared to the control group, patients with macular dystrophy had significantly poorer visual acuity, foveal MS, fixation MS, stability, and centrality, and more eccentric preferred retinal locations (PRLs). In macular dystrophy, the PRLs were most common on the superior side (48.3%). Compared to fixation in the atrophic region, PRLs out of the atrophic lesion gained more MS (7.41 vs. 0.89 dB, *p* = 0.001), although with less stable fixation (10.0 vs. 47.4%, *p* = 0.044). By multivariate linear regression, eccentric distance was found to be significantly associated with MS improvement (*p* = 0.023).

**Conclusions:**

The commonest location of PRLs in macular dystrophy is anatomically superior to the lesion. The dystrophic eye can gain better sensitivity by using PRLs outside the atrophic area.

## Introduction

Fixation behavior is an essential element for visual function and is an important element of visual performance, in addition to visual acuity. It is necessary to measure visual functions that are relevant for normal daily function; furthermore, reading problems are the main complaint of patients with maculopathies [1, 2]. Because maculopathies lead to loss of central vision, functional adaptations may occur and ultimately result in an enhancement of residual functional vision [3]. These patients often adopt an eccentric retinal area for fixation to regain reading ability after prolonged fixation attempts, and this preferred retinal location (PRL), as a “pseudofovea”, becomes the new center of the visual field [1, 4]. The scotoma shifts after the development of PRL, which contributes significantly to regaining reading ability and improving visual acuity [1, 5]. Information about the location and stability of the PRL is useful for clinicians in planning future treatment of patients with macular diseases and is essential for the correct interpretation of visual function and disease progression [6, 7].

Macular dystrophy is a progressive degeneration of retinal and/or choroidal tissue, which may be characterized by posterior pole dominant changes—a familial, bilateral, marked diminution of visual acuity, an early age of onset, a slow and progressive course, and no systemic physical or laboratory abnormalities [8]. Macular dystrophy affects visual function by decreasing visual acuity and destroys the retinal structure, leading to an atrophic appearance. For patients with maculopathy and central scotoma, the time course from onset of geographic atrophy to severe visual loss is usually several years [9, 10]. However, anti-retinal antibodies have been detected in some macular dystrophies or retinal degenerations, in which the disease progression may be different or altered by specific treatment [11, 12].

Microperimetry may be a useful clinical tool for assessing the location and stability of fixation while simultaneously measuring visual field sensitivity [6]. It has been used clinically to evaluate retinal sensitivity and its correlation to color fundus photos findings, optical coherence tomography (OCT), and fundus autofluorescence (FAF) [13]. It overlays the perimetric and fixation results on the fundus photograph and quantifies macular sensitivity and fixation pattern so that visual function can be correlated to retinal morphology; thus, the technique has the potential to improve our understanding for PRLs and visual sensitivity of macular diseases [6, 7].

Fixation behavior may provide valuable information to further understand visual performance in macular dystrophy and can be used as a functional parameter. The aim of this study was to investigate the functional changes (retinal sensitivity and fixation characteristics) as determined by microperimetry in patients with macular dystrophy and macular pucker.

## Methods

This retrospective, cross-sectional study was performed to evaluate patients with a clinical diagnosis of macular dystrophy or unilateral macular pucker from a single medical center in Kaohsiung, Taiwan, between 2008 and 2015. This study was carried out in adherence to the tenets of the Declaration of Helsinki and was approved by the institutional review board of Kaohsiung Chang Gung Memorial Hospital.

**Fig. 1:**
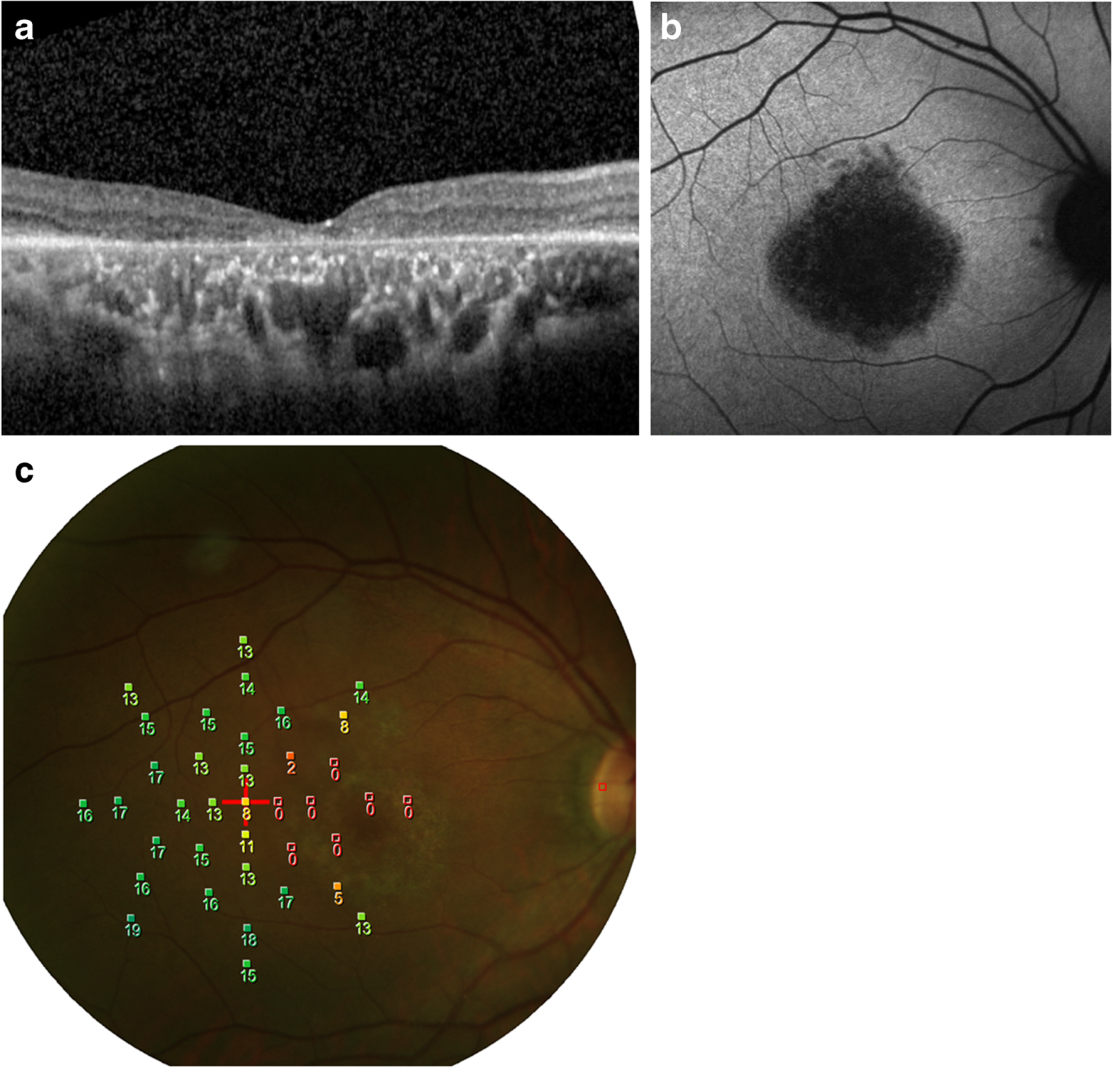
A case of clinical diagnosis of unclassified macular dystrophy with multimodal imaging, including **a** optical coherence tomography, **b** fundus autofluorescence, and **c** microperimetry

Based on history, symptoms, and fundus appearance, several modalities of diagnostic testing were utilized to achieve a correct diagnosis, including perimetry testing, OCT (Fig. 1a), FAF (Fig. 1b), fluorescein angiography, indocyanine green angiography, and electrophysiological testing. Clinical impressions of macular dystrophy were collected, including diagnosis of cone dystrophy, cone-rod dystrophy, Stargardt disease, and unclassified macular dystrophy. However, Best disease and juvenile foveoschisis were excluded due to extraordinarily better visual acuity and younger age, respectively. Patients with macular pucker were diagnosed based on OCT, with their fellow disease-free eyes being used as controls.

Best-corrected visual acuity (BCVA) and spherical equivalent (SE) were recorded for all patients. After pupillary dilation by 1% tropicamide and 10% phenylephrine hydrochloride, retinal microperimeter (MP-1, NIDEK Technologies, Italy) was used for the fixation test, microperimetry, and color fundus photographs (Fig. 1c). The right eye was tested first with fellow eye occluded, followed by the left eye. The fixation test provides information on the fixation position and stability of fixation; the machine parameters for defining fixation are presented in Table 1. The program “Medical” retina, as suggested by the operation manual, was used for microperimetry. Microperimetry measures retinal sensitivity in the central 20° diameter with 37 stimuli. The white background intensity was 4 asb (1.27 cd/m^2^), and a single cross was used as the fixation target. During the microperimetry, Goldmann III size stimuli with a duration of 200 ms were projected on the background. Stimulus intensity ranged from 0 dB (400 asb, 127 cd/m^2^) to 20 dB (4 asb, 1.27 cd/m^2^), with an initial intensity of 10 dB and light threshold strategy determined by using a 4–2 dB staircase strategy.

**Table 1:** Definition of fixation behavior in microperimetry

	Definition	Description
Centrality	Predominantly central	More than 50% of the fixation points located within the 2° diameter circle
	Poor central	More than 25% but less than 50% of the fixation points located within the 2° diameter circle
	Predominantly eccentric	Less than 25% of the fixation points located within the 2° diameter circle
Stability	Stable	More than 75% of fixation points located within the 2° diameter circle
	Relatively unstable	75% or more of the fixation points were located within the 4° circle, but less than 75% were within the 2° diameter circle
	Unstable	Less than 75% of the fixation points were located within the 4° diameter circle

The location of the foveola and the range of atrophic lesion were determined by the authors using FAF, which was performed by HRA (Heidelberg Retina Angiography, Heidelberg Engineering, Germany) under pupillary dilation. To elucidate the relation between fixation and atrophic area, FAF was compared with microperimeter by landmarks such as vessels and the optic disc. The disc diameter (DD) was defined as the average of the long and short axis diameters. Foveal and fixation mean sensitivity (MS) was defined as the average in the range of 1 DD from the center of the foveola and fixation point, respectively. MS improvement was the difference between foveal and fixation MS. The fixation eccentric distance was defined as the distance between foveola and fixation, measured as number of DDs.

Clinical and demographic characteristics of age, sex, the logarithm of minimal angle of resolution (logMAR) of BCVA, SE, foveal MS, fixation MS, difference between fixation and foveal MS, eccentric distance, direction, stability, and centrality of fixation were recorded and analyzed.

STATISTICS: Kolmogorov-Smirnov test for normality was used, as our data was not normally distributed. Continuous variables were expressed as medians (interquartile range, IQR), and Mann-Whitney *U* test and linear regression were used for univariate and multivariate analyses, respectively. Categorical variables were presented as percentages and a chi-square test with Bonferroni correction was used. Statistical significance was defined as a two-tailed *p* value of less than 0.05.

## Results

### Clinical characteristics of macular dystrophy

A total of 58 eyes of 58 patients were included: 29 left eyes of 29 patients in the macular dystrophy group and 29 normal eyes of 29 patients with unilateral macular pucker in the control group. The macular dystrophy group consisted of nine patients with cone dystrophy, seven patients with cone-rod dystrophy, seven patients with unclassified macular dystrophy, and six patients with Stargardt disease.

The macular dystrophy group consisted of 12 men and 17 women with a median age of 46.00 years (IQR 36.00–56.50 years, range 12–74 years). The median BCVA was 1.00 logMAR (IQR 0.70–1.30 logMAR, range 0–2.30 logMAR). The median SE was − 1.00 diopter (D) (IQR − 2.50 to 0.00 D, range − 7.38 to + 1.63 D). The median foveal MS was 0.00 dB (IQR 0.00–2.07 dB, range 0–10.50 dB), and the fixation MS was 1.08 dB (IQR 0.00–4.77 dB, range 0–16.23 dB). The difference between fixation and foveal MS was 0.00 dB (IQR 0.00–0.63 dB, range 0–16.23 dB). The median distance of the fixation point from the foveola was 0.50 DD (IQR 0.00–0.84 DD, range 0–3.09 DD). Twenty-three eyes (79.3%) in this group had developed eccentric fixation; the direction of PRL was superior in 14 eyes (48.3%), superior-temporal in 3 eyes (10.7%), inferior-nasal in 3 eyes (10.7%), temporal in 2 eyes (6.9%), and nasal in 1 eye (3.4%). Comparing the superior (14 eyes) and non-superior (9 eyes) shift on the PRL, no significant difference was noted between the groups with regard to median age (43.00 vs. 51.00 years, *p* = 0.528), BCVA (1.00 vs. 1.00 logMAR, *p* = 0.949), SE (− 1.00 vs. − 1.00 D, *p* = 0.680), foveal MS (0.45 vs. 0.00 dB, *p* = 0.557), fixation MS (3.46 vs. 9.38 dB, *p* = 0.449), MS improvement between fixation and fovea (0.77 vs. 3.80 dB, *p* = 0.656), and fixation eccentric distance (1.20 vs. 0.71 DD, *p* = 0.108).

In 19 eyes (65.5%), the fixation point was located within the atrophic lesion, while in the remaining 10 (34.5%) was located outside of the atrophic lesion.

### Comparison between macular dystrophy and control eyes

The clinical characteristics of the macular dystrophy and control groups are listed in Table 2. The median age in the macular dystrophy group was significantly lesser than in the control group (46.00 vs. 55.00 years, *p* = 0.014). The sex difference was not statistically significant, with 41.4% in the macular dystrophy and 20.7% in the control group being male (*p* = 0.155). The median BCVA was significantly poorer in the macular dystrophy group (1.00 logMAR) than in the control group (0.05 logMAR, *p* < 0.001). There difference in SE did not reach a level of significance: − 1.00 D in the macular dystrophy group and − 2.50 D in the control group, respectively (*p* = 0.185). The foveal MS in macular dystrophy (0.00 dB) was significantly lower than in the control group (18.76 dB, *p* < 0.001); the same was true for fixation MS as well (3.38 vs. 18.76 dB, *p* < 0.001). The MS improvement between fixation and fovea in macular dystrophy was also significantly higher in the macular dystrophy group (0.62 dB) than in the control group (0.00 dB, *p* < 0.001). The difference in fixation eccentric distance was also statistically significant in both the macular dystrophy (0.73 DD) and control groups (0.00 DD, *p* < 0.001). Both fixation stability (34.5 vs. 100%, *p* < 0.001) and centrality (17.2 vs. 100%, *p* < 0.001) were significantly lower in the macular dystrophy group.

**Table 2:** Clinical characteristics of the macular dystrophy and control groups

	Macular dystrophy	Control	*p* value
Number (eyes)	29	29	
Age (years old)	46.00 (36.00–56.05)	55.00 (50.5–63.0)	0.014*
Male (%)	12 (41.4%)	6 (20.7%)	0.155
BCVA (logMAR)	1.00 (0.70–1.35)	0.05 (0.00–0.15)	< 0.001*
SE (diopter)	− 1.00 (− 2.63–0.00)	− 2.50 (− 3.63–0.31)	0.185
Foveal MS (dB)	0.00 (0.00–2.71)	18.76 (17.54–19.69)	< 0.001*
Fixation MS (dB)	3.38 (0.23–9.46)	18.76 (17.54–19.69)	< 0.001*
MS improvement (dB)	0.62 (0.00–6.51)	0.00 (0.00–0.00)	< 0.001*
Fixation eccentric distance (DD)	0.73 (0.28–1.37)	0.00 (0.00–0.00)	< 0.001*
Fixation stability	10 (34.5%)	29 (100%)	< 0.001*
Fixation centrality	5 (17.2%)	29 (100%)	< 0.001*

Continuous variables expressed as median (interquartile range, IQR)

*BCVA* best corrected visual acuity, *logMAR* logarithm of minimal angle of resolution, *SE* spherical equivalent, *MS* mean sensitivity, *DD* disc diameter

*p < 0.05, statistically significant

### Fixation behavior of macular dystrophy—fixation in the atrophic lesion vs. out of the atrophic lesion

**Fig. 2:**
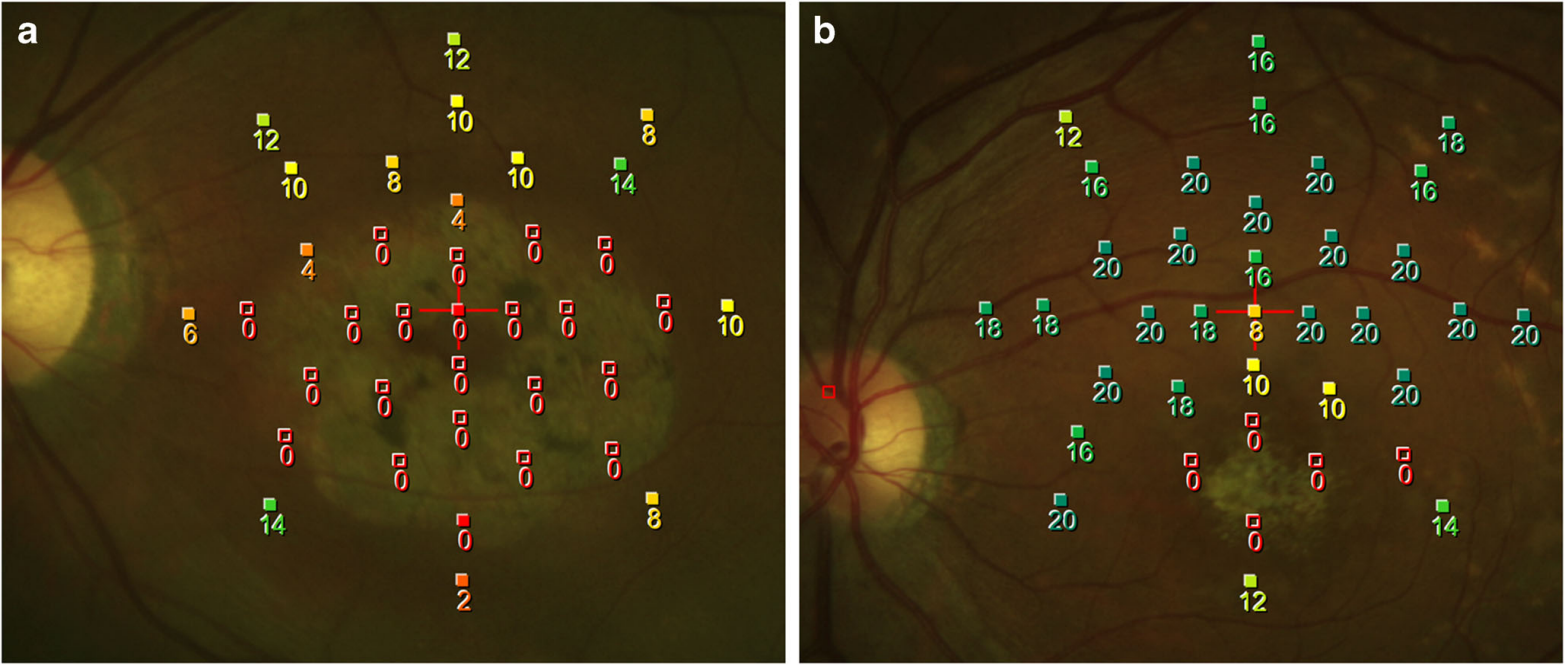
**a** An example of fixation located in an atrophic lesion with foveal mean sensitivity (MS) of 0 dB and fixation MS of 0.3 dB. **b** Another example of fixation located outside of the atrophic lesion with foveal MS of 2.4 dB and fixation MS of 14.4 dB

We further identified whether the PRLs were located within the atrophic lesion (Fig. 2a) or not (Fig. 2b). The comparison of PRLs located within or without of the atrophic lesion is presented in Table 3. A total of 19 eyes (65.5%) had the PRLs located within the atrophic lesion, while 10 eyes (34.5%) had a PRL located outside the atrophic lesion. None of the parameters of age, sex, BCVA, SE, foveal MS, and fixation centrality were significantly different between the sub-groups. The “fixation outside the atrophic lesion” group exhibited statistically significantly higher fixation MS (9.00 vs. 1.08 dB, *p* = 0.003), MS improvement between fixation and fovea (7.16 vs. 0.00 dB, *p* = < 0.001), and fixation eccentric distance (1.37 vs. 0.50 DD, *p* = 0.006), but a decreased stability (10.0 vs. 47.4%, *p* = 0.044).

**Table 3:** Comparison of fixation in or out of the atrophic lesion in macular dystrophy patients

	Fixation located in atrophic lesion	Fixation located out of atrophic lesion	*p* value
Number (eyes)	19 (65.5%)	10 (34.5%)	
Age (years old)	46.00 (26.00–58.00)	46.50 (40.00–54.25)	0.982
Male (%)	6 (31.6%)	6 (60.0%)	0.236
BCVA (logMAR)	1.00 (0.70–1.30)	1.15 (0.56–1.52)	0.513
SE (diopter)	− 1.00 (− 2.50–0.00)	− 0.63 (− 3.81–0.13)	0.747
Foveal MS (dB)	0.00 (0.00–2.07)	0.46 (0.00–3.38)	0.521
Fixation MS (dB)	1.08 (0.00–4.77)	9.00 (6.21–12.44)	0.003*
MS improvement (dB)	0.00 (0.00–0.63)	7.16 (4.90–9.42)	< 0.001*
Fixation eccentric distance (DD)	0.50 (0.00–0.84)	1.37 (0.93–1.52)	0.006*
Fixation stability	9 (47.4%)	1 (10.0%)	0.044*
Fixation centrality	4 (21.1%)	1 (10.0%)	0.454

Continuous variables expressed as median (interquartile range, IQR)

*BCVA* best corrected visual acuity, *logMAR* logarithm of minimal angle of resolution, *SE* spherical equivalent, *MS* mean sensitivity, *DD* disc diameter

*p < 0.05, statistically significant

### Prediction of MS improvement with PRLs

Multivariate linear regression was used to identify which, among the factors of age, SE, BCVA, foveal MS, and fixation eccentric distance, was associated with MS improvement between fixation and fovea. Only fixation eccentric distance was significantly associated with MS improvement (coefficient 2.641, standard error of coefficient 1.086, *p* = 0.023), while the others were not (SE *p* = 0.078, age *p* = 0.414, BCVA *p* = 0.725, foveal MS *p* = 0.984). Through more eccentric PRLs, the dystrophic eye seemed to gain better sensitivity.

## Discussion

Current outcome measures used to quantify macular anatomy and visual function are visual acuity, retinal thickness, and fluorescein angiography, but these measures do not always provide a complete picture of visual performance [14]. Microperimetry, in combination with a fixation test, has gained increasing interest recently, because it offers many functional parameters to better understand macular disorders, including retinal sensitivity threshold, fixation location, and stability. The MP-1 microperimeter allows for a rapid, safe, noninvasive, accurate, repeatable, topographically specific examination of the retinal threshold in selected retinal areas [15, 16]. Compared with static perimetry, microperimetry is more sensitive in detecting visual field defects [17].

For early-stage maculopathy, microperimetry can detect subtle visual functional changes in age-related macular degeneration (AMD), which cannot be detected by visual acuity tests [13, 16, 18–21]. Microperimetry, in combination with OCT, might be considered as surrogate markers of retinal function in the early stages of AMD [21, 22]. In addition to evaluating the current stage of macular disorders, microperimetry can also provide information on disease progression and treatment efficacy [18]. These data might be relevant in predicting long-term functional prognosis [23]. A significant deterioration of retinal sensitivity has been reported in early and intermediate AMD, whereas fixation stability changes only in intermediate AMD over a long-term [23]. Microperimetric studies on fixation stability after treatment of macular diseases have shown a strong correlation between better fixation stability and improvement in visual acuity [14]. In our clinical practice, microperimetry is used for evaluating the more sensitive visual function of patients with maculopathies such as dystrophy, pucker, or hole.

In the present study, patients with macular dystrophy were younger and had poorer visual acuity, foveal MS, and fixation MS, a more eccentric fixation, and poorer stability and centrality, but better MS improvement than the control groups. There was no significant difference in sex and SE between the groups (Table 1). In macular dystrophy, patients with eccentric fixation (most commonly superior to the atrophic lesion) had relatively poor fixation stability. Furthermore, fixation located outside the atrophic lesion had better fixation MS but less stability (Table 2).

Because the control eyes were the fellow eyes of patients with unilateral macular pucker, the median age was greater than in the macular dystrophy group. Eyes with macular dystrophy had poorer visual acuity, foveal MS, and stability than control eyes, as expected. We focused on the results of fixation behavior and retinal sensitivity change in the present study.

Different disorders may show different preferences for directional shift of the fixation [6, 23–28]. Mori F and associates report that fixation points were to the nasal side in cone dystrophy [29]. In patients with Stargardt disease, different studies indicate that the fixation points shifted superior to the atrophic lesion [6, 24, 29–31]. The development of eccentric fixation in Stargardt disease has been previously demonstrated: initial central fixation with decreased sensitivity, followed by alternation between central and eccentric fixation, and finally constant eccentric fixation [31]. In our study, 42.6% macular dystrophy patients showed superior eccentric fixation. As has been mentioned in previous studies, different maculopathies may develop different shifting directions, but a shift superior to the atrophic lesion is the most common. We postulate that the PRL shifts superiorly because patients get used to focusing on near objects below the plane of primary gaze in life, such as during reading, and this results in gradual stimulation above the atrophic retina. This hypothesis can be supported by other studies which state that superior regions with corresponding inferior visual fields are thought to be important in daily activities [28]. To further support this, Sunness and colleagues noted that there was a greater frequency of reading rate in those whose eyes fixated superiorly with the scotoma [24].

With regard to fixation stability, it is stable when the fixation point does not shift and is unstable in eyes with shifting fixation [29]; further, unstable fixation is strongly associated with poor visual acuity [32, 33]. Three case reports of occult macular dystrophy demonstrated that microperimetry revealed loss of sensitivity at the fovea [34–36], while one case preserved central fixation [34], another showed relatively unstable fixation [35], and the third exhibited unstable fixation with an infero-nasal PRL [36]. In eyes with Stargardt disease, a dense central scotoma causes fixation shift and PRL eccentricity correlates negatively with fixation stability [29, 30]. In our study, although fixation located outside an atrophic lesion demonstrated less fixation stability, BCVA did not show a significant difference, which is compatible with a previous study [37].

There were some limitations to this study. First, because it was not a longitudinal study but a retrospective, cross-sectional one, the alteration of fixation behavior during the disease progression could not be illustrated, such as the timing of developing PRL and speed of PRL location change. Second, the onset and duration of macular dystrophy were not identified, and hence, the severity of disease was not consistent across the group. Additionally, the diagnosis was based on the clinical features, and there was a lack of genetic information. Third, we enrolled a relatively small sample of patients in the study. Fourth, the mean age of the control group was not the same as that of the macular dystrophy group. Finally, the learning effects in perimetry may also affect the results, which can be overcome by successive examinations until it reaches a plateau.

Further prospective and longitudinal studies with larger samples and a longer follow-up period are warranted to better understand changes in visual acuity and fixation, and may be useful for understanding different macular disorders. This study was designed for monocular fixation and lacks binocular coordination during fixation [38]. Thus, further studies utilizing binocular-fixation may give different results.

We would like to emphasize the importance of using microperimetry and fixation tests to evaluate fixation patterns in maculopathy to deliver useful clinical information on visual function. The current report demonstrates fixation behavior with a detailed description of macular dystrophy and control eyes. Furthermore, we showed that fixation eccentric distance significantly affected MS improvement. Further analysis revealed that fixation behavior was altered in relation to functional change, including location, stability, and MS changes. These findings suggest that microperimetry is worth exploring as a method for novel interpretations of macular dystrophy.

In conclusion, microperimetry can provide functional evaluation of fixation behavior in macular dystrophy, including fixation behavior and retinal sensitivity. Eyes with macular dystrophy are prone to develop superiorly eccentric PRLs. Using PRLs outside the atrophic lesion, the dystrophic eyes can gain better sensitivity.
